# Spirulina as a Key Ingredient in the Evolution of Eco-Friendly Cosmetics

**DOI:** 10.3390/biotech14020041

**Published:** 2025-05-30

**Authors:** Sergiana dos Passos Ramos, Monize Bürck, Stephanie Fabrícia Francisco da Costa, Marcelo Assis, Anna Rafaela Cavalcante Braga

**Affiliations:** 1 Department of Biosciences, Universidade Federal de São Paulo (UNIFESP), Santos 11015020, Brazil; sergiana.passos@unifesp.br (S.d.P.R.); stephanie.costa@unifesp.br (S.F.F.d.C.); marcelotassis@gmail.com (M.A.); 2 Department of Physiology, Escola Paulista de Medicina (EPM), Universidade Federal de São Paulo (UNIFESP), São Paulo 04023062, Brazil; monize.burck@unifesp.br; 3 Department of Chemical Engineering, Universidade Federal de São Paulo (UNIFESP), Diadema 09913-030, Brazil

**Keywords:** microalgae, health, innovation, personal care, antioxidant activity

## Abstract

*Limnospira* spp., commercially known as spirulina, is widely recognized for its remarkable benefits due to its rich composition of bioactive compounds like phycobiliproteins, carotenoids, and phenolic compounds. These natural bioactive compounds not only serve as colorants but also offer potent antioxidant, anti-inflammatory, immunomodulatory, anticancer, antimicrobial, and anti-aging properties. As a result, spirulina and its components are increasingly used in cosmetic formulations to promote skin hydration, reduce wrinkles, and protect against UV radiation damage. Its bioactive components enhance fibroblast growth, boost collagen production, and prevent premature skin aging by inhibiting enzymes responsible for elastin degradation. Additionally, spirulina-based cosmetics have demonstrated wound-healing properties without genotoxic effects, with formulations containing C-phycocyanin particularly effective in shielding skin cells from UV-induced apoptosis. Despite these well-established benefits, there remains significant potential for the cosmetic industry to harness spirulina’s capabilities further. Research into the molecular mechanisms underlying its bioactive compounds in cosmetic formulations is still in its early stages, offering many opportunities for innovation. Emerging fields of biotechnology, such as nanotechnology and biocosmetics, could enhance the stability, efficacy, and delivery of spirulina-based ingredients, unlocking new possibilities for skin protection and rejuvenation. Furthermore, its proven biological properties align perfectly with the increasing consumer demand for safe, sustainable, and nature-inspired skincare solutions.

## 1. Introduction

Cosmetics are products applied to the human body for various purposes, including cleansing, healing, sun protection, antimicrobial effects, anti-aging, anti-cellulite treatment, moisturizing, beautification, perfuming, or altering appearance—all while ensuring no harm to human health. Millions of people use these products daily to maintain hygiene, enhance well-being, and improve their quality of life in pursuing beauty [1]. Natural ingredients and vitamins are vital in promoting cell renewal and protecting light-exposed skin, especially on the face [2]. On the other hand, synthetic ingredients commonly found in conventional cosmetics, such as coloring agents, have raised concerns. These compounds may trigger allergic reactions, particularly in individuals with dermatological sensitivities [3]. Beyond skin irritation, specific synthetic components are associated with broader health risks, toxicological issues, and environmental concerns [4,5,6], highlighting the growing importance of safer, more sustainable alternatives in cosmetic formulations.

Research on bioactive compounds derived from natural sources has gained significant traction in the cosmetic industry, driven by the growing demand for healthier, sustainable, safe, and eco-friendly products. This trend has emphasized the feasibility of using aquatic organisms, including marine and freshwater species, as valuable ingredients for personal care formulations Reviews conducted by Wang et al. [1], Mellou et al. [7], Morocho-Jácome et al. [8], Zhuang et al. [9], Hernández Muñoz et al. [10], and Mourelle et al. [11] have summarized various renewable marine organisms suitable for cosmetics, highlighting a diverse range of ingredients with unique beneficial properties, as well as the economic interest in this field. Among these organisms, microalgae and cyanobacteria stand out due to their rich metabolic diversity and bioactive compounds, particularly natural pigments like carotenoids, chlorophylls, and phycobiliproteins [8]. These pigments not only add color but also offer health-promoting benefits, including antioxidant and anti-inflammatory activities [12,13].

Of the many algae studied for cosmetic use, spirulina offers distinctive advantages compared to other marine and freshwater species. Its high protein content, abundance of essential amino acids, and rich supply of bioactive compounds—especially phycocyanin, a unique blue pigment with potent antioxidant and anti-inflammatory properties—make it well-suited for skin regeneration, photoprotection, and anti-aging applications. Unlike many macroalgae that require specific marine conditions, spirulina is highly adaptable to diverse cultivation systems, including low-cost, sustainable setups, and it produces stable pigments with proven efficacy. While other algae also contribute beneficial compounds such as polysaccharides and carotenoids, spirulina’s metabolic profile and functional versatility are more extensively documented and technologically exploited. Recent reviews on patent trends confirm that spirulina is gaining ground in cosmeceutical innovation, not only due to its efficacy but also for its compatibility with advanced delivery systems like nanoemulsions and encapsulation technologies, further enhancing its appeal for high-performance, natural cosmetic formulations [10,11].

Among cyanobacteria, *Limnospira platensis*, previously known as *Arthrospira platensis* and commercialized as spirulina, has become the most widely used cyanobacterium for animal and human nutrition. Its popularity stems from its substantial nutritional value. Several strains of spirulina have been certified by the Food and Drug Administration (FDA) as Generally Recognized as Safe (GRAS) owing to their lack of harmful effects on human health [14]. Spirulina can thrive in extreme environmental conditions, such as high alkalinity, salinity, and temperature, making it adaptable to various cultivation methods, including wastewater reuse in open and closed batch systems [15,16,17].

Although historically classified under the genus Spirulina, more recent morphological and molecular studies have shown that commercially cultivated strains exhibit distinct genetic and structural characteristics. This led first to their reclassification as *Arthrospira* and, more recently, to the definition of a new genus, *Limnospira*. The taxonomic revision aims to reflect the monophyletic nature of the cultivated lineages and their differences from the original type species. This reclassification has direct implications for the standardization of scientific nomenclature, regulatory frameworks for derived products, and the direction of future research [18,19,20]. The validity of this new classification has been reinforced by approaches combining genomic, morphological, and ecological data, which reveal phenotypic traits and growth patterns specific to *Limnospira*. This scientific redefinition enhances taxonomic accuracy and supports the development of safer and more effective biotechnological applications [21,22].

In recent decades, spirulina has garnered attention in academic research, focusing on its production [23], scale-up processes [24], and its bioactive compounds [25,26,27,28]. Additionally, research has focused on its application in food products [29,30,31]. Despite these advancements, original studies examining spirulina and its derivatives in cosmetic formulations remain sparse, particularly in product development for cosmetics and hair care [32]. Although the potential of spirulina for cosmetic applications is well-supported due to its well-documented composition and free radical scavenging abilities, there is a notable gap in understanding its interactions with other formulation components. Existing research has yet to thoroughly investigate spirulina’s molecular interaction with cosmetic vehicles, its physical properties, its spreadability, its stability, the release rates of its active compounds, and its clinical effects when applied in biomass or byproduct forms. Further research addressing these aspects could unlock new opportunities for incorporating spirulina into innovative and effective cosmetic products.

More recently, a review published by Ragusa et al. [33] highlighted spirulina as a promising ingredient for cosmetic formulations due to its anti-aging, revitalizing, remineralizing, and moisturizing properties for skin and hair. Similarly, Ikeda et al. [33] explored the interaction between spirulina biomass and its derivatives in dermatology, covering studies conducted on mice, in vivo and in vitro research, gene expression analysis, and cell proliferation. However, both reviews emphasized the scarcity of clinical studies on spirulina’s efficacy, and neither explored the integration of spirulina with emerging technologies like nanotechnology, 3D bioprinting, or biocosmetics development.

This work aims to bridge that gap by focusing on the current applications of spirulina and its components in cosmetics and beauty-related products, emphasizing their advantages and outcomes in the personal care industry. Furthermore, it aims to identify recent scientific findings and address future trends, particularly in cosmetics, where natural ingredients like spirulina are increasingly paired with cutting-edge technologies to meet consumer demand for innovative, sustainable products. Understanding the full potential of spirulina in cosmetics will require further research into its molecular mechanisms and interaction with emerging cosmetic technologies.

## 2. Material and Methods: Bibliographic Data Analysis

A search was conducted in the Elsevier Scopus database (https://www.scopus.com/, accessed in 1 January 2025) to understand the critical aspects of spirulina biomass in biocosmetics and other beauty-related products through the search term ((((“Spirulina” AND “platensis” OR “phycocyanin” OR “c-phycocyanin” OR “biomass” OR “residual biomass”) AND (“biocosmetics” OR “cosmetics” OR “dermatology” OR “rinse off” OR “leave on” OR “makeup” OR “beauty”)))), and it returned 124 documents. The search term was determined so that the maximum number of studies related to cosmetics and spirulina could be obtained for the investigation.

**Figure 1:**
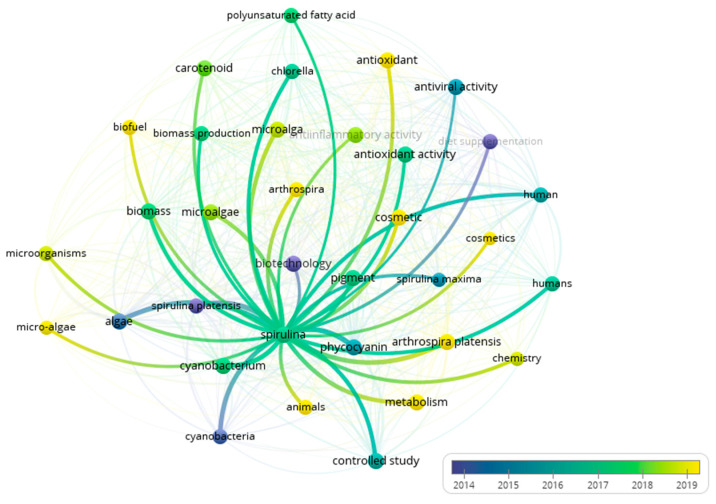
Bibliometric network visualization map. The co-occurrence of terms that encompass spirulina and beauty-related products. The software automatically set the overlay color range (2014–2024) to illustrate the terms better.

These findings composed the bibliometric overlay visualization map (Figure 1). It was done over VosViewer (software version 1.16.19, Leiden University, Leiden, the Netherlands). The sizes of the circles and labels are directly dependent on the weight; that is, the greater the occurrence of the term, the larger the dimensions of the circles and labels will be. The closer circles are more frequently published together, and the default colors range from blue (previous years) to green to yellow (last years), which are shown in the bottom right corner of the visualization [27]. Therefore, “Spirulina” was related to “biotechnology” and “diet supplementation” around 2014, and more recently, “Spirulina” is more related to “cosmetics.” This well-founded observation can justify the lack of studies linking spirulina to cosmetics since it is an emerging field of study. Nevertheless, the application of spirulina has gained a wide variety for several industries, such as pharmaceutics, food, and feed and biofuels, once more evidencing its potential to be explored and the possibility of cooperation between industries, when considering the use of byproducts in different sectors.

As exclusion criteria on the Elsevier Scopus database, the type of document (limited to articles) and the years of publication were chosen, which comprised the period from 2013 to 2024. Thereby, 57 articles were collected. Interestingly, only 15.78% of this total were original articles studying specific properties of spirulina and its application in cosmetics. Further studies based on personal care products were included from the initial readings. As a result, the most relevant studies for this review were covered and comprised the following sections.

## 3. Spirulina Production and Recovery

Production is an essential point for any industry. From this point of view, information regarding the pros and cons of spirulina as a candidate in the biocosmetics sector regarding production, upstream and downstream, must be considered without underestimating the importance of critical points like scale-up, productivity, and economy. Spirulina represents 30% of the 10,000 tons of dry-weight algal biomass produced globally in the microalgae industry [34]. Upstream processes can be considered microalgae and cyanobacteria research’s baseline. These activities directly impact biomass quality and quantity, making them commercially and technically essential. They also evolve processes such as bioreactor design, culture medium preparation, CO_2_ supplementation, and environmental factor adjustment and control [35]. Besides the cultivation medium, photobioreactors for microalgal culture have been examined for design and operation. The main pillars of the upstream process in microalgae research include different cultivation modes, photobioreactor design, culture media preparation, microalgae supply, environmental factors, and microalgal growth monitoring [35].

**Figure 2:**
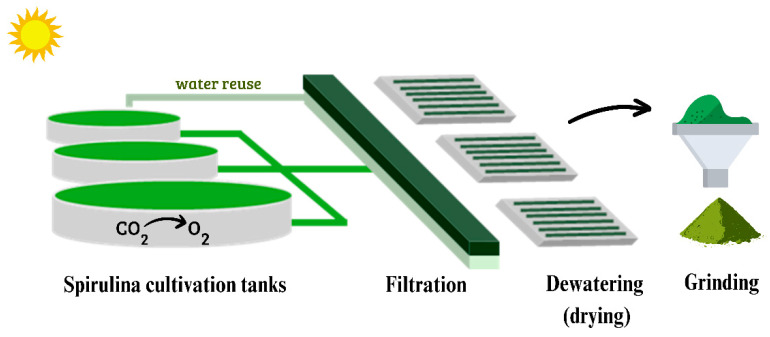
Spirulina biomass production process: cultivation, harvesting, filtration, grinding and crushing, and environmental benefits (water reuse and carbon footprint reduction).

Spirulina is primarily grown in low-cost, easy-to-build, and maintained open ponds. These systems have low biomass yield, high evaporation rates, and difficulties maintaining ideal culture settings and are susceptible to contamination. Spirulina is less susceptible to contamination as a cyanobacterium that grows at increased pH (9.5 to 11.0). The productivity of biomass and other metabolites with biotechnological interest depends on the cultivation medium. Three media from the literature were selected in this study: Zarrouk’s, Hiri’s, and Jourdan’s. Spirulina production is affected by medium nitrogen concentration and nitrogen source. Light intensity significantly affects spirulina production, interfering with any photosynthetic organism [36]. Harvesting and dewatering are needed to remove biomass from culture media. The literature reports using coagulants to reduce membrane fouling and increase filtration flow, improving microalgae harvesting. Besides, the first step in downstream processing is cell disruption, which involves breaking the cell wall and membranes to release high-value components from the cytoplasm and internal organelles [37,38,39]. Figure 2 summarizes the spirulina cultivation, harvesting, and handling process.

After the products are liberated from the cells, several extraction and fractionation methods must be utilized to separate proteins, lipids, and carbohydrates from the biomass. All remaining components must be functional. Separating and purifying extracted products yields specified products. Purification includes distillation and membrane filtering [39]. All those steps will depend on the application of the spirulina and its components as ingredients; each added step increases the cost, so comprehending the added value of each potential ingredient will also determine the final feature of the cosmetic formulated with these components.

The sustainability of spirulina stands on an environmental, economic, and social basis. The cultivation process using wastewater and photobioreactors has gained environmental notoriety since it does not depend on soil, as agriculture usually does [16]. In this sense, semiarid climatic conditions and uncommon cultivable soils could fit its production, also generating wealth and employment. Furthermore, spirulina cultivation requires fewer areas compared to conventional vegetable cultivation farms and in closed photobioreactors, which can double the biomass concentration every 2–5 days. Therefore, replacing synthetic and natural assets derived from vegetables with more sustainable and profitable cultivation fields is also a sustainable alternative [40].

Spirulina cultivation requires CO_2_ for photosynthesis, contributing to reducing the carbon footprint and global warming. The acquisition of this green technology will allow many companies to reduce their carbon footprint and effluent production, achieving environmental sustainability and reducing the economic cost of these companies. Besides, in photosynthesis, O_2_ is released into the atmosphere, thus benefiting the environment [41]. A drawback in spirulina cultivation is the high cost of the chemical-based culture medium due to their high nutritional value. Therefore, alternative forms have been evaluated to reduce the cost of this chemical-based culture medium, and wastewater is a promising alternative nutrient source. Wastewater is enriched with nitrogen and phosphorus, making replacing the compositions in the chemical-based culture medium possible [42].

Different wastewaters, such as industrial, aquaculture, and domestic, could be used as a substrate for spirulina cultivation after less complex filtration, dilution, and supplementation treatments. Although prior treatments for wastewater are necessary, using wastewater as a cultivation medium for spirulina can solve part of the environmental pollution problems and achieve a sustainable circular economy [41].

The upscaling of spirulina cultivation entails considerable technical complexities, notably in achieving effective contamination control and maintaining uniform quality at an industrial scale. Nevertheless, spirulina represents a strategic and environmentally sustainable alternative for protein production, offering a solution with a markedly lower ecological impact than conventional sources. Promoting the advancement of cultivation technologies specifically adapted to regional climate and market conditions is essential to ensure spirulina’s commercial viability and promote it as an active ingredient in cosmetic formulations considered environmentally friendly [43].

Spirulina’s unique characteristics and potential for sustainable production make it a promising alternative to traditional crops and animal products in terms of minimizing carbon emissions and promoting food security. Besides, spirulina cultivation presents a significantly more sustainable option for reducing the carbon footprint and mitigating global warming compared to conventional crops. Unlike these land-intensive crops, which require fertile arable land and substantial freshwater inputs and are often associated with deforestation and ecosystem disruption, spirulina can be grown in closed photobioreactors on marginal, non-arable land with minimal freshwater use and without reliance on climatic conditions. Its rapid growth rate, exceptionally high protein content (up to 70%), and dense nutritional profile—surpassing that of beef in key micronutrients—make it a highly efficient food source. Additionally, spirulina production generates markedly lower greenhouse gas emissions, especially when compared to livestock systems and the input-heavy cultivation of feed crops like maize and sugarcane. These advantages position spirulina as a technologically advanced, resource-efficient, and climate-resilient alternative that can meaningfully contribute to a sustainable food future [44,45,46].

## 4. Spirulina and Its Components: Biological Effects

*Limnospira platensis* and *Limnospira maxima*, previously named *Spirulina platensis* and *Spirulina maxima,* are the best-known species [47]. Cultivation usually takes place at a pH greater than 9.5, which inhibits the growth of contaminating microorganisms [48] in a simple production process with significant economic impact, where spirulina’s byproducts can originate biofuels [17] biodegradable food packaging [49,50,51], the source for food and feed [52,53,54,55,56,57], and health and pharmaceuticals [27], thus mitigating waste and contributing to the reduction of greenhouse gas emissions. Therefore, it is considered a highly sustainable resource [33].

Spirulina is a cyanobacterium capable of transforming solar energy into chemical energy. Its cultivation occurs on photobioreactors or opened systems with wastewater [17,58,59]. The acquired biomass is rich in proteins (for about 60%), carbohydrates (24%), and fatty acids (7%) [60], in addition to vitamins and phycobiliproteins, namely C-phycocyanin (C-PC), allophycocyanin, and phycoerythrin [61]; carotenoids; and chlorophylls and phenolic compounds, in other words, bioactive compounds of great interest for industrial application due to antioxidant activity. The blue pigment C-PC is usually the target one due to its vast majority and antioxidant activity [12], acting as a free radicals’ scavenger that leads to anti-inflammatory, immunomodulatory [26], anticancer [27,62], and antimicrobial effects [63].

Regarding cosmetics application, the antioxidant activity is related to anti-aging; preventing and reducing the formation of wrinkles, expression lines, and stretch marks [1,64]; hydration and oil control of the skin [65]; and reducing the harmful effects of UV radiation, protecting the skin and hair [66]. As a natural ingredient derived from sustainable biomass, C-PC is an excellent antioxidant suitable as a colorant for biocosmetic formulations, notwithstanding their moisturizing, healing, anti-aging, whitening, and anti-acne actions [33].

Additionally, studies have shown that extracts containing isolated compounds from microalgae, such as proteins, vitamins, and fatty acids, promote biological effects similar to those obtained with pure biomass. These isolated compounds have demonstrated efficacy in various applications, both in health and cosmetics. For example, microalgal proteins can aid tissue repair and strengthening, while vitamins provide antioxidant properties and essential nutrients. Fatty acids, in turn, are known for their anti-inflammatory and moisturizing capabilities [67,68].

Various cosmetic products incorporating microalgae are already available on the market, offering proven benefits for skin and hair. These products ensure deep hydration, promoting skin and hair softness, strength, and shine. Additionally, spirulina contains a high concentration of C-FC, mycosporine-like amino acids (MAAs), and other substances that can help protect the skin from damage caused by free radicals and sun exposure. Thus, using microalgae in cosmetics enhances aesthetic appearance and contributes to the overall health of skin and hair [66,69].

Cutaneous aging brings concerns from both an aesthetic and clinical standpoint. Due to the chronological effect, the natural aging process is related to intrinsic factors. However, external factors, such as ultraviolet radiation, are responsible for skin aging. Both cases cause malfunctioning of cutaneous tissues, with degradation of essential components for skin health and integrity, such as collagen and elastin [70,71].

Spirulina has shown the ability to act on fibroblasts and keratinocytes, with the potential against types of premature aging, promoting cell proliferation, inhibition of enzymes responsible for premature aging, and antioxidant capacity, protecting the skin from oxidative stress and inflammation. In an in vitro study using a human dermal fibroblast cell line, the crude protein extract from spirulina biomass showed the ability to increase the levels of epidermal growth factor receptor (EGFR) while inhibiting the expression of matrix metallopeptidase 8 (MMP-8), enzymes related to premature aging. Furthermore, the extract promoted increased fibroblast growth and collagen production (42% and 142%, respectively, compared to the control) and inhibited enzymes responsible for elastin degradation [72].

A study evaluated the action of the ethanolic extract of spirulina against damage caused by UVB radiation. The extract reversed UV radiation-induced damage, such as cell viability and senescence, DNA damage, and destruction of skin fibroblasts. The optimized extracts were applied to normal human fibroblasts and exposed to UVB radiation. In vitro analyses demonstrated the inhibition of DNA damage caused by UVB radiation, such as pyrimidine dimers, as well as a significant reduction in cell cycle arrest and senescence in dermal fibroblasts through the decrease in the expression of MMP-1 and MMP-3 [73]. In the same line of investigation, Mapoung et al. [74] also assessed the phenolic extract of spirulina against UVB radiation, showing a high absorption capacity, registering a sun protection factor of 30. The same extract was applied to fibroblasts exposed to UVB radiation, reducing intracellular oxygen free radical levels (ROS) and inhibiting inflammatory cytokine synthesis (IL-6 and IL-8). The authors inferred that these biological effects are associated with the high concentration of phenolic compounds in spirulina biomass [74].

As previously mentioned, the blue pigment extracted from spirulina biomass is a valuable compound with high antioxidant action. It is responsible for a large part of the biological effects presented by cyanobacteria. C-PC was extracted from spirulina biomass, and its protective effects against UVB radiation were evaluated. Human dermal fibroblast and epidermal keratinocyte cell lines were incubated with C-PC for 24 h, and their action against UVB-induced apoptotic cell death was assessed. The results indicate that treatment with phycobiliprotein increased the expression of heme-oxygenase-1, a protein associated with combating oxidative stress. Furthermore, the treatment increased the expression of anti-apoptotic factors while reducing pro-apoptotic factors and blocked chromatin condensation and DNA fragmentation induced by UVB radiation [75]. Thus, in addition to spirulina biomass, isolated compounds such as C-PC can protect skin cells against the apoptotic effects of ultraviolet radiation.

Few studies have explored the biological effects of spirulina or its blue pigment incorporated into cosmetic formulations. Gunes et al. [76] developed a body cream incorporated with spirulina extract, and its cytotoxic, genotoxic, and wound-healing effects were evaluated in vitro. The skin cream, including 1.125% *Spirulina platensis* crude extract, showed a more significant wound-healing effect on keratinocyte cell lines and greater cell viability. The creams showed no genotoxic effects on human peripheral blood cells. In addition, collagen production was increased in cell lines treated with skin cream incorporated with spirulina extract at the same concentration mentioned earlier [76].

**Figure 3:**
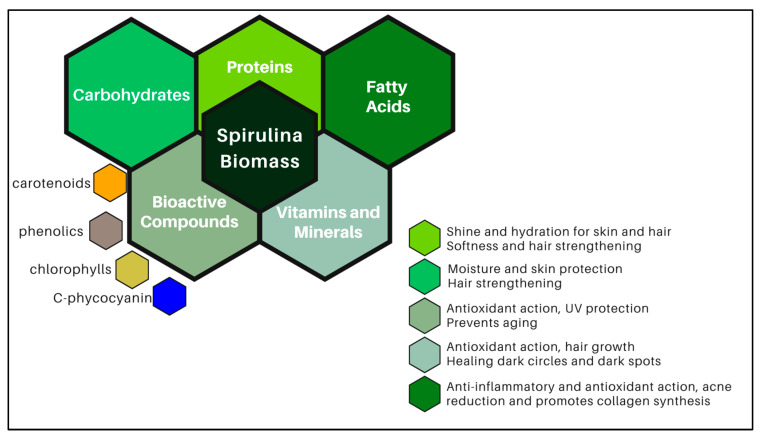
Compounds present in spirulina biomass and their main biological effects related to skin and hair health.

Based on the provided text, spirulina and its compounds, such as C-PC, demonstrate promising anti-aging effects and skin protection. In vitro studies indicate that spirulina can promote cell proliferation, inhibit enzymes responsible for premature aging, increase collagen production, protect against damage caused by UV radiation, and benefit wound healing. This suggests great potential for using spirulina and its compounds in cosmetic formulations. Figure 3 connects the main compounds in spirulina biomass and describes their biological effects on skin and hair health.

## 5. Application of Spirulina in Personal Care Products

Microalgae and cyanobacteria present a significant number of components with industrial potential. However, they are understudied and undervalued. Marine, freshwater, and terrestrial species number in the thousands, and this large number of species inherently includes biochemical compound diversity. The biochemical potential for economic exploitation is countless, significantly because environmental factors change the biochemical composition and produce bioactive compounds, increasing the obtaining of different metabolites according to the variation of the cultivation parameters and conditions. They differ categorically (microalgae are eukaryotic, and cyanobacteria are prokaryotic). Nevertheless, both perform photosynthesis, and cultivation concepts and problems coincide and are comparable regarding industrial potential [77].

A common point of interest is the non-toxicity of spirulina and its derivatives for topical applications with remarkable coloring capacity. As color is a critical feature when choosing products, the health claim about its natural and renewable source leads to high value and innovation. Positively, spirulina biomass was incorporated into cosmetic formulations without significantly modifying rheological parameters [33]. It should be mentioned that the possibility of using dried or aqueous extract solution is an excellent strategy for better handling with physical properties, which is an advantage.

In the specialized literature, much is said about phytochemical complexes, carbohydrates, fatty acids, and phenolic compounds, but no recent study has focused on applying them. For elucidation, phenolic compounds from spirulina encapsulated as liposomes presented antifusarium properties with slower release than phenolics without encapsulation [78]. Still, to the present, it has not been clinically tested, even though liposomes are excellent vehicles for delivering active ingredients to the deeper layers of the skin.

Predominantly, the studies did not specify what “Spirulina extract” means, whether commercially or laboratory obtained. For example, the dried extract of Karray et al. [79] and the resuspended spirulina extract applied by Infante et al. [80] seem to be C-PC, but it was not characterized. None of the summarized studies below (Table 1) have evaluated the purity of the active ingredient. Moreover, a stability test was carried out at 50 °C, an elevated temperature that does not necessarily emulate the actual daily storage of the product, and instability was identified after two weeks. Table 1 uses the nomenclature for spirulina as presented by the original authors of each publication. This approach preserves the context of the original studies. It highlights the variability in taxonomic classification found throughout the literature, despite the current academic consensus, based on recent studies, that considers the genus *Limnospira* more appropriate.

In addition, when the temperature was 4 °C, the authors observed blue exudate in the creams loaded with C-PC and moringa in contrast to the stability in C-PC and pomegranate formulation after three weeks [79]. The authors concluded that pomegranate protected spirulina from fermentation, but the microbiological test did not indicate contamination; therefore, further analyses should be carried out to understand the stability of the product.

Among cosmetics for daily use, shampoo is one of the most frequently used formulations [4]. The study by Silva et al. [81] proved the effectiveness of hair formulations made with 0.1% spirulina extract in strengthening the hair fiber, reducing combability, and increasing the shine of the strands. Resourcefully, the biodegradable composite developed by Adli et al. [82] is highly creative since it brings together a film for personal care (e.g., face or hair mask) and a way of rapidly delivering C-PC through a thin layer—economically appealing since it is an expensive bioproduct [10].

Besides the need for clinical studies, more sensory acceptance analysis must be done. Sensorial acceptance plays a crucial role in marketing, and just Delsin et al. [65] and Infante et al. [80] conducted a sensory evaluation with more than ten volunteers. Delsin et al. [65] also evaluated the group for a considerable time (28 days) and compared the groups by age.

**Table 1:** Personal care products formulated with spirulina or its constituents.

Cosmetic Product	Organism	Bioproduct	Association	Results	Reference
Hair dye	*Arthrospira platensis*	Phycocyanin (0.15 g)	Ascorbic acid and Arabic gum	Temporary hair dye with good physical stability	[83]
Cosmetic for skin	*Arthrospira platensis*; *Tetraselmis* sp.; *Dunaliella* sp.	Whole biomass (0.5, 1.5, and 2.5%)	Oil-in-water commercial emulsion	0.5% spirulina, 1.5% *Tetraselmis* sp., and 2.5% *Dunaliella* sp. received significant major sensory evaluation scores. Spirulina cream presented the greatest antioxidant activity	[84]
Cosmetic cream	*Arthrospira platensis*	Dried extract	Emulsifiers, essential oils, vitamin E, pomegranate peel, or moringa leaves	Spirulina and pomegranate cream had organoleptic characteristics preserved during the evaluation. The formulation was stable at 4 °C for 3 weeks.	[79]
Sunscreen	Spirulina	Commercial dried extract (0.1% *w*/*w*)	Commercial sunscreen and dimethylmethoxy chromanol	Nonallergenic stable formulation with a significant increase in skin net elasticity and viscoelasticity. The skin-lightening effect was observed. SPF * was nearly 30.	[85]
A topical formulation for skin and hair	*Spirulina maxima*	Dried extract (0.1%)	Tapioca starch; corn starch; PEG-75 lanolin	Film forming was successfully obtained for skin and hair protection. The formulation decreased transepidermal water loss and could protect the hair against daily damage. Spirulina did not affect the rheological parameters.	[80]
Dermocosmetic	Spirulina	Spirulina extract (0.1% *w*/*w*)	Gel-cream	Mature skin presented more hydration than younger skin; oil control for both ages; it was not related to dermal thickness alongside 28 days.	[65]
Cream for the treatment of acne vulgaris	*Spirulina platensis*	Freeze-dried powder (5 g)	Oil-in-water creams with nonionic emulgents	The formulation containing sucrose ester and spirulina showed antimicrobial properties and the highest antioxidant activity, besides nontoxic effects when used for skin treatment	[86]
Biodegradable composite for cosmetics	Spirulina	Commercial phycocyanin	Polylactic acid and alginate powder	Nontoxic for human fibroblasts (<1000 µg·mL^−1^). Phycocyanin/alginate, with a 40/60 ratio, was the best for the active layer of the film, with the best flexibility and release.	[82]

* Sun Protection Factor

### 5.1. Biocosmetics

To understand the evolution of biocosmetics in the world industry, it is necessary to know that it is linked to the awareness of a portion of the population of the harmful effects of some substances that were used in conventional cosmetics, which, many times, caused hypersensitivity of the dermis, dermatitis, among other allergic reactions [87]. The first biocosmetic prominence took place in the 70s, when cosmetology was in evidence in scientific and social movements as they sought alternatives that did not bring environmental damage or human health, so natural and organic cosmetics emerged as an alternative to meet the needs of personal well-being without all the problems related to chemical substances that have adverse health effects that are present in conventional cosmetics [88].

Biocosmetics is a term used for natural cosmetics mainly consisting of organic ingredients, free from chemical fertilizers and free substances such as dyes and synthetic fragrances, silicones, and petroleum derivatives. The bioproduct must also not contain anything of animal origin and must not be tested on them. In the 1990s, the cosmetics industry in Brazil and worldwide began to adapt to new consumers willing to pay more for natural and organic cosmetics, considering the entire production chain with a view towards an ecological and sustainable process, preserving the environment and the quality of life of future generations [89]. Additionally, biocosmetics are produced from 100% natural ingredients, free of pesticides and chemical fertilizers in the cultivation of raw materials, using techniques related to green chemistry in the extraction and production processes. The market for natural organic cosmetics has increased significantly, and the projection is for greater demand for these products [90]. Due to the increase in the consumer market and the need to develop formulations using compounds less aggressive to health and the environment, researchers are turning to discover natural actives that provide effective action in skin and hair treatment, increasing the action potential of substances already used or leading to modifications in the forms of obtaining and processing and the delivery system of natural actives [87].

### 5.2. Hair Cosmetics and the Composition of Shampoos

Shampoos are an essential and probably the most used hair product, developed to clean leather and hair. In antiquity, soaps were used for this purpose, and the first shampoo only came into existence less than a century ago, around 1933; with the advancement of shampoo technology, its chemical composition can range from 10 to 30 ingredients with different functions, which can be classified as (i) cleaning agents, (ii) conditioning agents, and (iii) active ingredients and additives. The biological function of hair is to protect the scalp, but it also plays an essential role in self-esteem and self-perception of beauty [3]. The scalp is one of the most absorbent parts of the body; since the ingredients contained in hair formulations fall directly into the bloodstream, it is vital to assess their safety for our body [4,5].

For cleaning to occur, the component responsible for performing this function is a surfactant or detergent. These are anionic surfactants, molecules with an amphoteric structure capable of binding to aqueous and fat-soluble components, removing dirt and excess sebum from the hair [3]. Foam is not synonymous with cleanliness, but it is one of the factors that most attract consumers when choosing shampoo. Secondary surfactants or co-surfactants can increase foam, such as betaines, among which the best known is cocoamidopropyl betaine, usually obtained in a 30% aqueous solution [86].

Currently, the requirements for a good shampoo go beyond its ability to foam and clean; it is necessary to maintain the health of the strands [4] without causing dryness and allergic reactions. Frequent surfactant exposure can cause skin dryness and irritation [65]. Among the primary surfactants, sodium lauryl sulfate (SLS), widely found in shampoos, soaps, and products related to skin cleansing, has more significant irritant potential due to its high cleaning potential, which can provide irritability in the same proportion and can cause long-term damage such as dryness [5], skin peeling, hair growth problems, and allergies [91].

The term “sulfate-free” is designated to products free of this type of surfactant, the sulfates. This category of products has emerged in the last two decades due to consumer concerns about alkyl sulfate and alkyl ether sulfate products and their possible skin and eye irritations [92]. Among them, sodium isethionate (SCI) has wide application in personal care products, especially in bar products, with cleaning and foaming, due to its compatibility with the skin, softness, and emollient properties. It is essential to consider that the pH of shampoos must be close to the natural value of the hair and scalp (5.5–6.0) [5]; unlike soaps, shampoos with alkaline pH can cause damage to the hair. The study by Jeraal et al. [93] reveals that the high activity of SCI at neutral pH improves its skin compatibility, thus reducing the effects of negative charges compared to soaps and alkyl sulfates. Additionally, SCI is derived from a renewable source, produced on a large scale from the hydrolysis of coconut oil, obtaining a mixture of carboxylic acids—between octanoic acid (C8) and octadecanoic acid (C18). Therefore, it is a more natural and ecological alternative to conventional surfactants.

Conditioning agents are also applied in formulations to impart softness, increase manageability and shine of the hair, balance the negative static charges of cleaning agents, and reduce frizz [3]. Some of these conditioning components are fatty alcohols (for example, cetyl alcohol and stearyl alcohol), vegetable oils, and butter [3,5], which are applied with an emollient action and offer nutrition to the hair. In addition to surfactants and conditioning agents, active ingredients and additives are also used in shampoo formulation. While additives generally provide color and aroma, active ingredients, with activities such as antioxidant action and increased hydration, are included in formulations that benefit the hair [5]. The study by Castro et al. [32] presented the formulation of three solid shampoos containing 1% (*w*/*w*) C-PC, 5% (*w*/*w*) spirulina biomass and 5% (*w*/*w*) spirulina residual biomass (obtained after C-PC extraction). The physical-chemical characterization produced results within the ranges established by Brazilian legislation for all evaluated parameters. According to the microbiological analysis results, all samples are safe and sanitary. All pigmented shampoos had higher antioxidant activity and phenolic compound content than the control shampoo, and the formulation made with spirulina biomass had more excellent stability in its pigmenting action, as did the antioxidant activity when the two methods were studied in the evaluation of this parameter during the evaluated period.

More recently, a study used isolated proteins extracted from spirulina for application in hair treatment cream. The treatment was carried out for 9 days, and the results indicate that the formulation containing the spirulina protein isolate increased hair moisture and hydration, in addition to maintaining the integrity of the hair cuticles through the increase in keratin, resulting in stronger hair and offering a better overall appearance [88].

## 6. Market and Consumer Behavior Toward Environmentally Friendly Cosmetics

The awareness of people’s health has increased from the food to the cosmetics industry, making the market more receptive to natural cosmetics. This is evidenced by the growing use of natural materials and resources in manufacturing cosmetics, driven by the marketing appeal of these products. This trend is fueled by the ever-increasing environmental and health awareness worldwide, especially in developed countries. Producers and consumers of cosmetics are adapting to this new reality. The popularity of natural cosmetics can be attributed to the growing perception of the adverse effects of synthetic additives on health and the environment. As a result, health consciousness, ecological motivation, and environmental knowledge are essential to understanding green buying behavior [90].

Many cosmetics contain ingredients derived from marine bioproducts. Their cosmetics use can be attributed to their skin benefits [94]. Extracts from algae moisturize, improve blood circulation, activate cell renewal and metabolism, regenerate tissues, have anti-inflammatory effects, and increase skin resistance [95,96]. In particular, the production of natural cosmetics with spirulina extract can have a significant positive impact on the cosmetic industry. Spirulina extract can replace the chemical ingredients present in most cosmetics, as it contains a wide variety of components, such as proteins, carbohydrates, lipids, vitamins, minerals, and natural pigments, such as chlorophyll, carotenoids, and C-phycocyanin [97,98]. Additionally, spirulina has several biological activities of interest, such as antioxidant, anticancer, and anti-inflammatory activities, which add value to cosmetics production. Cosmetics with spirulina have the potential to be a promising product for the industry due to the richness of biological and chemical benefits, as well as being low-cost and environmentally friendly.

Due to the increasing access to information about the current global environmental situation, cosmetics consumers increasingly demand product additives. As a result, manufacturers are expanding their range of natural cosmetics to take advantage of the changing consumer attitude. This is making the global industry highly competitive and raising the level of excellence of its products. The eco-friendly strategy is now critical to business sustainability, but only some companies can implement it. The lack of regulation in the current market is also a barrier to implementing natural cosmetics, increasing consumer distrust [99]. Therefore, the introduction of legal regulations should be intensified to fill this gap. In addition, new marketing strategies can attract consumers who purchase eco-friendly cosmetics and new consumers to the sector.

For consumers of natural cosmetics, the relationship between price performance and quality are essential factors to consider. If the product does not meet the consumer’s expectations, the purchase becomes a negative experience. A study by Amberg and Fogarassy [100] analyzed the profile of Hungarian consumers regarding natural cosmetics, categorizing them into three groups: one group that prefers to buy natural cosmetics, another that prefers chemical cosmetics, and a third group that purchases both. It was found that these groups have different perceptions about the benefits of natural cosmetics, with some determining factors including price, knowledge about the cause, and brand tradition in the market, among others. In a similar study, Matić and Puh [101] analyzed the profile of Croatian consumers. They concluded that those more likely to buy organic food and care about their health are more open to using natural cosmetics. In another work, a study on the consumption of natural cosmetics in South Africa, Shimul et al. [102] emphasize that empowering consumers through education and learning is fundamental to motivating or convincing them to make eco-friendly purchases. Based on these observations, it is evident that the socioeconomic characteristics of the region, as well as access to information, are crucial factors for the maintenance and acceptance of natural products in cosmetic formulations.

Regarding using algae as natural additives in cosmetics, Lafarga et al. [103] observed a remarkable need for more knowledge about the fundamental aspects of microalgae. This demonstrates that although microalgae are considered sustainable, nutritious, healthy, and safe additives, there is still little information on this subject. Another problem with using algae extracts is the poor exploration of these extracts as primary active ingredients. This is still a largely unexplored field in the cosmetics industry.

**Figure 4:**
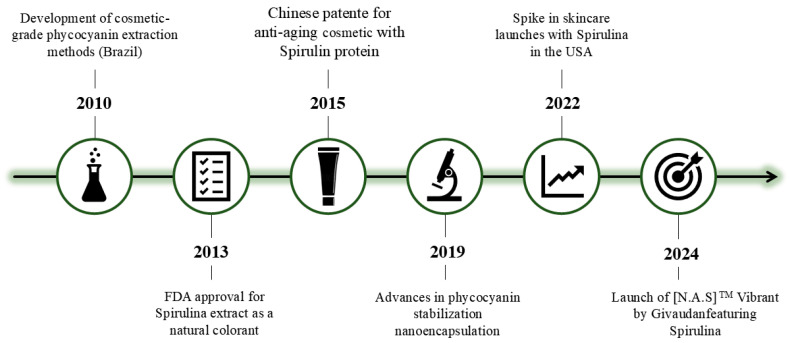
Historical timeline of spirulina’s emergence in the cosmetic industry.

To enhance the contextual understanding of spirulina’s emergence in the cosmetic industry, a historical timeline is presented in Figure 4. This timeline highlights pivotal moments in the biotechnological, regulatory, and commercial development of spirulina-based cosmetic products. Key milestones include establishing extraction techniques for phycocyanin, stabilization and delivery systems advances, landmark patents, international approvals, and the market introduction of notable cosmetic formulations. Together, these events illustrate the progressive incorporation of spirulina into modern cosmeceutical innovations and validate its growing commercial relevance.

There is a growing trend in the use of microalgae in the cosmeceutical industry, incorporating products extracted from their biomass into cosmetic formulations. However, consumers will not choose natural cosmetic products if they are less effective than chemical ones. Additionally, basic knowledge about natural additives, environmental awareness, and a preference for natural products do not always create a positive association for consumers. As spirulina and algae, in general, are not common ingredients in the Western diet, the perception of their benefits must be clear so that products that use spirulina as an additive are better accepted and consumed.

In line with this increasing interest in natural and algae-based cosmetics, several companies worldwide have launched commercial products incorporating spirulina as an active ingredient. These products vary in type and application, ranging from facial care and haircare to nutritional supplements with cosmetic appeal. The table below presents a selection of companies currently offering spirulina-based cosmetic products, highlighting their country of origin, product category, intended application, and purchase links. Table 2 illustrates the growing integration of spirulina into the global cosmetics market and provides practical examples that support its commercial viability and consumer appeal.

## 7. Future Trends in the Application of Nanotechnology and Spirulina in Cosmetic Formulations

Despite the biotechnological advances in the use of spirulina, significant gaps remain in understanding the molecular mechanisms underlying the activity of its biomass constituents. Elucidating these mechanisms is essential for a comprehensive understanding of the biological effects attributed to its bioactive compounds and for the safe expansion of its cosmetic applications. Moreover, comprehensive studies on cutaneous toxicity and allergenic potential are critical to ensure topical formulations containing spirulina’s long-term safety and efficacy. Cutaneous permeation also stands as a key limitation, as the effectiveness of the active compounds relies on their ability to traverse the epidermal barrier [33,90].

Integrating nanotechnology and bioactive compounds derived from spirulina represents an emerging and innovative approach to developing more effective and functional cosmetic products. These bioactive compounds can be incorporated into nanomaterials to enhance their stability, bioavailability, and controlled release, which are key features for topical and dermatological applications. Moreover, using biomass or biopolymers from spirulina to fabricate bioactive nanostructures and scaffolds offers advantages such as biocompatibility and reduced risk of rejection while maintaining their biological activity during encapsulation and release processes [88].

Among the emerging technologies, producing nanofibers via electrospinning using natural pigments, particularly phycocyanin extracted from spirulina, has shown considerable promise. Studies have demonstrated that polymeric nanofibers containing phycocyanin exhibit high encapsulation efficiency, thermal stability, and pH-responsive behavior. These characteristics are highly relevant for designing smart delivery and protective systems for bioactive cosmetic formulations. The irreversible color change observed in phycocyanin-containing nanofibers further indicates the structural integrity and stability of the encapsulated compounds, which are highly desirable attributes in topical products exposed to environmental stressors [49,50].

The fabrication of nanofibers incorporating natural pigments also presents significant potential in sensory and functional applications. The use of spirulina as a pigment source enabled the development of nanofibers with tunable optical and thermal properties, which may be exploited in cosmetic products as stability indicators or active sensors. Electrospinning, due to its ability to produce fibers with a high surface area-to-volume ratio and adjustable morphology, is a versatile tool for creating systems that enhance the interaction between bioactive agents and human skin [50].

Another promising strategy involves the incorporation of spirulina in extruded starch-based matrices, which significantly influences the nano and microstructural organization of these materials. The formation of amylose-lipid complexes induced by intracellular compounds of spirulina during extrusion led to the development of foams with increased thermal stability and controlled porosity. These structural attributes can be translated into cosmetic applications, especially for formulating structured vehicles (gels or aerogels) that demand specific rheological and mechanical properties to ensure effective topical delivery of active ingredients [104,105].

Lastly, using spirulina as a reducing agent in the green synthesis of metal nanoparticles has introduced a novel and sustainable avenue for functional material development. A recent study demonstrated the successful synthesis of cerium oxide nanoemulsions using spirulina extract, with superior antioxidant, antimicrobial, and selective cytotoxic effects compared to non-encapsulated nanoparticles. The potential application of such systems in cosmetics includes multifunctional formulations with antipollution, anti-aging, and skin-brightening properties. Nanoencapsulation further enhances these benefits, improving efficiency and environmental stability. Furthermore, the green synthesis approach aligns with current trends in sustainable chemistry and circular economy, which are increasingly valued by the contemporary cosmetic industry [105].

## 8. Conclusions

Applying spirulina in cosmetic formulations has many benefits, but much is still to be explored. Despite the great proven potential of the spirulina biomass and the C-PC pigment extracted from this cyanobacterium, few studies have evaluated its potential to produce biocosmetics, opening a series of opportunities for product development in the academic and industrial fields. Considering the survey conducted in this research, it is noteworthy that C-PC is used not only as a natural pigment but also as a promoter of several health benefits mainly related to antioxidant action. Furthermore, spirulina and its derivatives are potential natural ingredients to replace synthetic preservatives and antioxidants, which are widely associated with several harmful effects when used in the short and long term. Technological advances in micro- and nanoencapsulation have become essential tools to overcome key limitations related to spirulina-based compounds’ chemical instability and low skin permeability. These delivery systems protect sensitive bioactive compounds from degradation and enhance their bioavailability, controlled release, and penetration into deeper skin layers, significantly boosting spirulina’s effectiveness and commercial viability in modern cosmetic formulations. However, this technology can lead to an increase in the added value of the final product and, consequently, low demand due to its high cost. In this sense, marketing strategies should be employed to make natural products popular and convey their various environmental and human health benefits.

## Figures and Tables

**Table 2:** Cosmetic products with spirulina—brands and product overview.

Brand/Company	Product Type	Application	Origin	Purchase Link
Tulípia	Facial cream	Skincare	Goiânia, Brazil	https://tulipia.com.br/ (accessed on 24 May 2025)
Sallve	Anti-oil stick	Skincare	São Paulo, Brazil	https://www.sallve.com.br/ (accessed on 24 May 2025)
Mad 4 Life	Skincare kits	Nutrition and skincare	Cajamar, Brazil	https://www.mad4.life/ (accessed on 24 May 2025)
GYADA Cosmetics	Haircare line with Spirulina	Hair strengthening	Lagerhaus Tankstelle, Austria	https://www.ecco-verde.com/ (accessed on 24 May 2025)
SutaCosmetic	Serums, creams, facial products	Anti-aging, hydration	Estoril, Portugal	https://sutacosmetic.com/ (accessed on 24 May 2025)
MySpirulina Cosmetics^®^	Creams, facial products	Skincare	Reinbek, Germany	https://www.ocean-pharma.de/en/ (accessed on 24 May 2025)
SkinOwl	Body concentrate with spirulina	Hydration and skin brightening	Boise, USA	https://www.skinowl.com/ (accessed on 24 May 2025)
EmerginC	Facial toner with spirulina	Toning and pH balance	New York, USA	https://emerginc.com/ (accessed on 24 May 2025)
Golde	Powder facial masks with spirulina	Soothing and nourishment	New York, USA	https://golde.co/ (accessed on 24 May 2025)

## Data Availability

No new data were created or analyzed in this study.

## Abbreviations

The following abbreviations are used in this manuscript:

FDA	US Food and Drug Administration
GRAS	Generally Recognized as Safe
EGFR	epidermal growth factor receptor
MMP-8	matrix metallopeptidase 8
C-PC	C-phycocyanin
ROS	reactive oxygen species
SLS	sodium lauryl sulfate
SCI	sodium isethionate
